# Clinical and pathologic features of primary membranous nephropathy in Turkey: a multicenter study by the Turkish Society of Nephrology Glomerular Diseases Working Group

**DOI:** 10.1080/0886022X.2022.2079526

**Published:** 2022-07-04

**Authors:** Abdulmecit Yildiz, Sena Ulu, Aysegul Oruc, Ali Riza Ucar, Savas Ozturk, Selma Alagoz, Necmi Eren, Ismail Kocyigit, Simal Koksal Cevher, Ali Burak Haras, Abdullah Sumnu, Turgay Arinsoy, Garip Sahin, Gultekin Suleymanlar, Caner Cavdar, Gizem Kumru Sahin, Ilhan Kurultak, Abdulkadir Unsal, Gulizar Sahin, Sinan Kazan, Erhan Tatar, Mehmet Dıkec, Belda Dursun, Hayriye Sayarlioglu, Kultigin Turkmen, Ayse Serra Artan, Nimet Aktas, Zulfikar Yilmaz, Ahmet Behlul, Hamad Dheir, Sim Kutlay, Nurhan Seyahi

**Affiliations:** a Department of Nephrology, Med Fac, Uludag Univ, Bursa, Turkey; b Department of Nephrology, Med Fac, Afyon Kocatepe Univ, Afyon, Turkey; c Department of Nephrology, Istanbul Fac Med, Istanbul Univ, Istanbul, Turkey; d Department of Nephrology, Haseki Training and Res Hosp, Istanbul, Turkey; e Department of Nephrology, Cerrahpasa Med Fac, Istanbul Univ, Istanbul, Turkey; f Department of Nephrology, Med Fac, Kocaeli Univ, Kocaeli, Turkey; g Department of Nephrology, Med Fac, Erciyes Univ, Kayseri, Turkey; h Department of Nephrology, Ankara Numune Training and Res Hosp, Ankara, Turkey; i Department of Nephrology, Dr Lutfi Kirdar Kartal Training and Res Hosp, Istanbul, Turkey; j Department of Nephrology, Med Fac, Medipol Univ, Istanbul, Turkey; k Department of Nephrology, Med Fac, Gazi Univ, Ankara, Turkey; l Department of Nephrology, Med Fac, Eskisehir Osmangazi Univ, Eskisehir, Turkey; m Department of Nephrology, Med Fac, Akdeniz Univ, Antalya, Turkey; n Department of Nephrology, Med Fac, Dokuz Eylul Univ, Izmir, Turkey; o Med Fac, Ibni Sina Hosp, Ankara Univ, Ankara, Turkey; p Department of Nephrology, Med Fac, Trakya Univ, Edirne, Turkey; q Department of Nephrology, Hamidiye Sisli Etfal Training and Res Hosp, Istanbul, Turkey; r Department of Nephrology, Sultan Abdulhamit Han Res and Training Hosp, Istanbul, Turkey; s Department of Nephrology, Bozyaka Training & Res Hosp, Izmir, Turkey; t Department of Nephrology, Bakirkoy Sadi Konuk Training and Res Hosp, Istanbul, Turkey; u Department of Nephrology, Med Fac, Pamukkale Univ, Denizli, Turkey; v Department of Nephrology, Med Fac, Sutcu Imam Univ, Kahramanmaras, Turkey; w Department of Nephrology, Meram Med Fac, Necmettin Erbakan Univ, Konya, Turkey; x Department of Nephrology, Med Fac, Bezmialem Vakif Univ, Istanbul, Turkey; y Department of Nephrology, Med Fac, Dicle Univ, Diyarbakir, Turkey; z Department of Nephrology, Burhan Nalbantoglu State Hosp, Nicosia, Cyprus; aa Department of Nephrology, Med Fac, Sakarya Univ, Sakarya, Turkey

**Keywords:** Histopathology, immunofluorescence, primary membranous nephropathy, nephrotic syndrome, Turkey, kidney biopsy

## Abstract

**Background:**

We aimed to evaluate the features of primary membranous nephropathy (MNP) in Turkish people.

**Methods:**

This is a retrospective analysis of patients with biopsy-proven primary MNP. We obtained the data collected between 2009 and 2019 in the primary glomerulonephritis registry of the Turkish Society of Nephrology Glomerular Diseases Study Group (TSN-GOLD). Patients with a secondary cause for MNP were excluded. Clinical, demographic, laboratory, and histopathological findings were analyzed.

**Results:**

A total of 995 patients with primary MNP were included in the analyses. Males constituted the majority (58.8%). The mean age was 48.4 ± 13.9 years. The most common presentation was the presence of nephrotic syndrome (81.7%) and sub nephrotic proteinuria (10.3%). Microscopic hematuria was detected in one-third of patients. The median estimated glomerular filtration rate (eGFR) was 100.6 mL/min/1.73 m^2^ (IQR, 75.4–116.3), and median proteinuria was 6000 mg/d (IQR, 3656–9457). Serum C3 and C4 complement levels were decreased in 3.7 and 1.7% of patients, respectively. Twenty-four (2.4%) patients had glomerular crescents in their kidney biopsy samples. Basal membrane thickening was detected in 93.8% of cases under light microscopy. Mesangial proliferation and interstitial inflammation were evident in 32.8 and 55.9% of the patients, respectively. The most commonly detected depositions were IgG (93%), C3 complement (68.8%), and kappa and lambda immunoglobulin light chains (70%). Although renal functions were normal at presentation, vascular, interstitial, and glomerular findings were more prominent on biopsy in hypertensive patients. No significant effect of BMI on biopsy findings was observed.

**Conclusions:**

Despite some atypical findings, the main features of primary MNP in Turkey were similar to the published literature. This is the largest MNP study to date conducted in Turkish people.

## Background

Membranous nephropathy (MNP) is the leading cause of idiopathic nephrotic syndrome worldwide [1]. During the last decade, significant improvements have highlighted the pathophysiology of MNP with the determination of target autoantigens. With this regard, the nomenclature and classification of MNP are rearranged. Around 80% of primary MNP cases result from pathogenic IgG4 antibodies targeting the podocyte antigen phospholipase antigen-2 receptor (PLA2R). In addition, Anti-THSD7A antibodies were also shown as the pathogenic cause of glomerular injury in 3–5% of patients of primary MNP [2]. Recently, laser microdissection and mass spectrometry methods revealed new antigens and their respective antibodies in patients with MNP [3].

Pathologically, MNP is characterized by glomerular basal membrane thickening and subepithelial *in situ* immune complex deposition in the glomerular capillary wall. Glomeruli are usually spared from inflammatory cell infiltration and mesangial immune deposits. Immune fluorescein (IF) is almost all positive for IgG and C3 staining. Less often, C5b-C9, IgA, and IgM are positive in IF [4]. The staining for PLA2R and THSD7A by IF or with immunohistochemistry contributes additive finding to identify the classification of MN [3]. Significant staining besides IgG and C3, such as Ig M, mesangial expansion, and inflammation, are the secondary MNP suggestive features.

Nephrotic syndrome is the main clinical presentation with a rate of 80% in patients with primary MNP [5,6]. However, clinical features can vary from asymptomatic proteinuria to full-blown nephrotic syndrome among patients according to the stage of the disease [3,7]. Clinical presentation is usually similar for secondary MNP.

The prevalence of MNP in Turkey was stated as 25.6% among 3875 primary glomerulonephritis patients by Turkmen et al. [8]. However, this study was an epidemiologic study and did not report clinical and pathologic features of MNP in detail. Thus, we aimed to perform the largest epidemiological study to date investigating demographic characteristics, laboratory values, and kidney biopsy features of patients with primary MNP under the auspices of the Turkish Society of Nephrology in Turkey.

## Methods

### TSN-GOLD primary glomerulonephritis registry

This is a retrospective, multicenter, cross-sectional study of patients with primary MNP between 2009 and 2019 (Figure 1). The data were obtained from a prospectively maintained database, the Primary Glomerulonephritis Registry of the Turkish Society of Nephrology Glomerular Diseases Study Group (TSN-GOLD) [9]. Forty-seven nephrology units throughout Turkey have been providing data for the registry, including kidney-biopsy proven glomerular diseases. Previously, several studies have been conducted with the data obtained from this registry [8,10,11]. The total number of patients in the registry at the time of the evaluation was 4399. However, we analyzed the data of 3875 adult patients after excluding patients without light and/or immunofluorescence microscopy findings. Patients with negative IF but markedly thickened basement membranes were not excluded. Patients with a histopathologic diagnosis of MNP were evaluated for secondary causes. Each patient was screened for malignancy according to the clinical practice of each center. Routine physical examination was performed in order to detect lymphadenopathy, organomegaly, breast mass, skin lesion, or urological abnormality. Eligible patients had lung imaging for lung cancer (chest X-ray or computed tomography [CT]). Abdominal imaging (ultrasound or magnetic resonance imaging or CT) and endoscopic scans were made for abdominal origin cancers. Breast and gynecological cancers were screened by cervical smear, mammography, and breast ultrasonography for eligible female patients as well. Also, standard laboratory testing was performed in order to exclude secondary forms of glomerulopathy, including protein electrophoresis, serum light chains, viral serology, complement level, and soluble rheumatologic markers, such as ANCA profile, antinuclear antibody. Patients with suspected genetic disease; eye examination, audiogram, and also genetic screening in some centers [9]. A total of 995 of 3875 adult patients (25.7%) were registered as primary MNP and included in this study.

The study design was approved by the Ethics Committee of Istanbul Medical Faculty of Istanbul University, Turkey.

### Data collection

Demographic data included age, gender, body mass index, chronic comorbid conditions, and systolic and diastolic blood pressure measurements at the presentation were recorded. The average of at least two systolic and diastolic blood pressure measurements was recorded. Laboratory findings including plasma glucose, serum urea, creatinine, albumin, uric acid, lipid profile, complement 3 (C3) and complement 4 (C4) levels, daily protein excretion, presence of pyuria, and hematuria at the time of biopsy were evaluated. To determine the amount of daily protein excretion, 24-h urine was collected. The estimated glomerular filtration rate (eGFR) was calculated *via* the Chronic Kidney Disease Epidemiology Collaboration (CKD-EPI) equation [12]. Clinical presentation and biopsy indications were evaluated. The clinical and laboratory findings were compared according to age, eGFR. All the data were obtained from the electronic database system of the TSN-GOLD.

### Renal biopsy evaluation

MNP was diagnosed based on the percutaneous kidney biopsy in this study. Light microscopy and immunofluorescence microscopy evaluations were performed in all pathologic specimens, but electron microscopic evaluation was performed in only a minority of biopsy materials because of the difference in the conditions between the centers. For light microscopic evaluation, paraffin-hidden tissue was examined under hematoxylin-eosin, periodic acid–Schiff, aldehyde fuchsin orange G, and periodic acid-silver methenamine stains.

The fluorescence intensity of immunoglobulins (IgG, IgA, and IgM) and complement components (C3 and C1q) was assessed by means of a semi-quantitative scale ranging between 0 and +3. Zero meant negative staining, whereas +1, +2, and +3 correspond to weak, moderate, and strong staining, respectively. The number of totals, global sclerotic and segmental sclerotic glomeruli, the presence of thickening of the basal membrane, mesangial proliferation, interstitial inflammation, and tubular atrophy were evaluated for each biopsy sample.

Of 928 patients who underwent IF evaluation, 64 had insufficient IF staining due to sclerotic glomeruli. However, these patients were considered as MNP because of the significant thickening of the basement membrane and the absence of serological and biopsy findings of other pathologies.

### Definitions

MNP was diagnosed by kidney biopsy, in which light microscopy showed diffuse thickening of the glomerular basement membrane throughout all glomeruli without significant hypercellularity, and presence of subepithelial ‘spikes’ on the capillary wall and positive granular staining of IgG and C3 in capillary walls in immunofluorescence microscopy [2]. In limited centers, electron microscopy evaluation supported MNP diagnosis with subepithelial immune deposits in the glomeruli.

In diabetic patients, patients with diabetic retinopathy or patients with typical diabetes-related glomerular lesions, such as nodular sclerosis or diffuse mesangial sclerosis were excluded.

In centers that could analyze serum anti-PLA2R levels, MNP diagnosis was confirmed with the positivity of anti-PLA2R. However, primary MNP was generally defined by excluding the aforementioned etiologies regardless of anti-PLA2R.

Nephrotic syndrome was defined as the presence of proteinuria (>3.5 g/d), low serum albumin level (<3.5 g/dL), and edema [13]. The presence of 5 or more white blood cells per high power field (HPF) in urine microscopic examination was defined as pyuria. Pyuria was deemed sterile when urine culture results revealed no bacterial growth or patients had no symptoms suggestive of urinary tract infection. The presence of five or more red blood cells per HPF in urine microscopic examination was defined as hematuria.

Nephritic syndrome was defined as the presence of subnephrotic proteinuria, hematuria, oliguria, and hypertension (HT). In addition, we classified a patient with a nephrotic syndrome with nephritic characteristics as mixed nephrotic syndrome.

## Statistical analysis

We used the SPSS version 25.0 software package (IBM, Armonk, NY) to analyze the data of the study. The Shapiro–Wilk test, histogram, and Q–Q plot were used to test the normality of the variables. Normally distributed variables were presented as mean ± standard deviation, and non-normally distributed variables were given as median and interquartile range (IQR). The independent samples t-test and the Mann–Whitney U-test were used for numerical variables in two group comparisons in accordance with the normality status of the variables. The Chi-square test and Fisher’s exact test were used to compare the categorical variables between the groups. In comparing more than two groups, ANOVA or Kruskal–Wallis with the Bonferroni correction was used, appropriately. A *p* value < 0.05 was accepted as statistically significant.

## Results

### Clinicodemographic characteristics and laboratory findings

A total of 995 (58.8% male, mean age: 48.4 ± 13.9 years) patients with primary MNP were included in the analyses. The median follow-up time was 33 months (IQR, 10–70.3). The rate of HT was 33%, and diabetes mellitus was 11.1%. Of these, 24.9% of the study population was smokers.

The leading clinical presentation and thus indication for kidney biopsy was nephrotic syndrome with a rate of 81.7%. Asymptomatic urinary abnormalities, mainly subnephrotic proteinuria, mixed nephrotic syndrome, and nephritic syndrome, were the other clinical presentations and indications for kidney biopsy (10.3, 3.5, and 2%, respectively). Microscopic hematuria was detected in one-third of patients.

The laboratory findings revealed a median eGFR value of 100.6 mL/min/1.73 m^2^ (IQR, 75.4–116.3), mean serum albumin as 2.7 ± 0.8 g/dL, median daily protein excretion 6000 mg (IQR, 3656–9457). Serum C3 and C4 complement levels were decreased in 3.7 and 1.7% of patients, respectively. Anti-PLA2R antibodies were studied in only 138 (13.9%) patients. Of these, 98 (71.0%) patients were positive for anti-PLA2R antibodies. All positive patients underwent kidney biopsy, as well. The clinical, demographic, and laboratory parameters of the entire study population are presented in (Table 1).

**Table 1.** Clinicodemographic characteristics of the study population.

Parameters	*n*	Patients
Age (years)	980	48.4 ± 13.9
Sex		
Female	995	410 (41.2%)
Male		585 (58.8%)
Comorbidities		
Hypertension	964	343 (35.6%)
Diabetes mellitus	963	107 (11.1%)
Smoking status (current smoking)	566	141 (24.9%)
Pretibial edema	909	669 (73.6%)
Body mass index (kg/m^2^)	526	27 (15.81–51.42)
Indications of kidney biopsy	978	
* *Asymptomatic urinary abnormalities		101 (10.3%)
* *Nephritic syndrome		20 (2.0%)
* *Nephrotic syndrome		799 (81.7%)
* *Mixed Nephritic-nephrotic		34 (3.5%)
* *Other		24 (2.5%)
Systolic blood pressure (mm/Hg)	881	130 (13–210)
Diastolic blood pressure (mm/Hg)	881	80 (40–130)
Pyuria	877	115 (13.1%)
Hematuria	877	289 (33%)
Biochemistry		
* *Glucose (mg/dL)	863	92 (44–991)
* *Blood urea nitrogen (mg/dL)	929	15 (1–171)
* *Creatinine (mg/dL)	937	0.8 (0.2–11.1)
* *CKD-EPI estimated GFR (mL/min/1.73 m^2^)	930	100.6 (3.9–191.6)
* *Uric acid (mg/dL)	816	5.7 (2–11.9)
* *Total cholesterol (mg/dL)	793	277 (88–721)
* *Triglyceride (mg/dL)	802	201 (44–999)
* *HDL-cholesterol (mg/dL)	741	51 (14–149)
* *LDL-cholesterol (mg/dL)	764	176.50 (14–799)
* *Total protein (g/dL)	825	5.2 (2.8–10.2)
* *Albumin (g/dL)	899	2.7 (0.9–5.6)
* *Alanine aminotransferase (U/L)	820	16 (5–219)
* *Calcium (mg/dL)	823	8.6 (5.5–15)
* *Hemoglobin (g/dL)	890	13.2 ± 1.9
* *Hematocrit (%)	867	39 (22–86)
* *Proteinuria (mg/d)	838	6000 (3–31,185)
* *Sedimentation rate (mm/h)	654	43 (1–140)
Anti-phospholipase A2 receptor antibody		
* *Negative	138	40 (29%)
* *Positive		98 (71%)
C3 complement		
* *Decreased	722	27 (3.7%)
* *Normal		695 (96.3%)
C4 complement		
* *Decreased	706	12 (1.7%)
* *Normal		694 (98.3%)
Duration of membranous nephropathy (month)	992	29 (0–347)

CKD-EPI: Chronic kidney disease epidemiology study; GFR: glomerular filtration rate.

The data were expressed as mean ± standard deviation, median (minimum: maximum), and *n* (%).

### Kidney biopsy findings

MNP was diagnosed based on the renal biopsy findings. The median number of glomeruli per kidney biopsy samples was 16 (min–max, 1–98). The presence of one or more globally sclerotic glomeruli was frequent among patients with a rate of 50%, whereas only 14.5% of the patients had segmental glomerular sclerosis.

In light microscopic examination, the most common finding was increased capillary basal membrane thickening (93.8%). Mesangial proliferation and interstitial inflammation were evident in 32.8 and 55.9% of the patients, respectively. Almost half of the entire study population had interstitial fibrosis and tubular atrophy.

In microscopic immunofluorescence evaluation, the most commonly detected depositions were IgG (93%), C3 complement (68.8%), and kappa and lambda immunoglobulin light chains (70%). Details of light and immunofluorescence microscopy findings are summarized in Table 2. Electron microscopic examination was performed in only 2.1% of the patients.

**Table 2.** Histopathologic findings of kidney biopsies.

Parameters	*n*	Patients
Histopathology, *n* (%)		
Mesangial proliferation	929	305 (32.8%)
Subendothelial immune complex deposition	793	33 (4.2%)
Subepithelial immune complex deposition	809	332 (41%)
Endocapillary proliferation	589	33 (5.6%)
Exudative glomerular changes	555	25 (4.5%)
Increased capillary basal membrane thickness	968	908 (93.8%)
Interstitial inflammation	941	526 (55.9%)
Interstitial fibrosis		
Absent		515 (54.4%)
Grade 1 (<25%)	946	324 (34.2%)
Grade 2 (25–50%)		82 (8.7%)
Grade 3 (>50%)		25 (2.6%)
Vascular changes	946	323 (34.1%)
Tubular atrophy		
Absent		501 (52.6%)
Grade 1 (<25%)		374 (39.3%)
Grade 2 (25–50%)	952	59 (6.2%)
Grade 3 (>50%)		18 (1.9%)
Number of glomerular changes, median (min–max)		
Total glomeruli in biopsy sample	974	15 (1–98)
Global sclerotic	913	1 (0–24)
Segmental sclerotic	825	0 (0–18)
Crescentic glomeruli	816	0 (0–10)
Cellular crescents	584	0 (0–3)
Fibrocellular crescents	584	0 (0–3)
Fibrous crescents	584	0 (0–3)
Immunofluorescence microscopy findings, *n* (%)	928	
IgG		
Negative		64 (7%)
(+)	915	72 (7.9%)
(++)		200 (21.9%)
(+++)		579 (63.3%)
IgM		
Negative		671 (74.6%)
(+)	899	152 (16.9%)
(++)		56 (6.2%)
(+++)		20 (2.2%)
IgA		
Negative		756 (83.7%)
(+)	903	101 (11.2%)
(++)		30 (3.3%)
(+++)		16 (1.8%)
C3		
Negative		281 (31.2%)
(+)	901	259 (28.7%)
(++)		206 (22.9%)
(+++)		155 (17.2 %)
C1q		
Negative		716 (88.4%)
(+)	810	63 (7.8%)
(++)		18 (2.2%)
(+++)		13 (1.6%)
Kappa		
Negative		148 (30.4%)
(+)	487	90 (18.5%)
(++)		123 (25.3%)
(+++)		126 (25.9%)
Lambda		
Negative		146 (30%)
(+)	486	90 (18.5%)
(++)		124 (25.5%)
(+++)		126 (25.9%)
Fibrinogen		
Negative		677 (92.7%)
(+)	730	37 (5.1%)
(++)		11 (1.5%)
(+++)		5 (0.7%)

The data were expressed as median (minimum: maximum) and *n* (%).

### Comparison of patients according to presence hypertension at presentation

The patients were grouped according to the presence of HT and obesity at presentation. HT was defined as a previous diagnosis of HT and obesity, defined BMI over 30 kg/m^2^ at the time of biopsy. In MNP patients with HT, interstitial inflammation, fibrosis, vascular change, and sclerotic glomeruli were more prominent. No difference was found between hypertensive and normotensive MNP patients with regard to protein excretion (Table 3). Diabetes and HT rates were significantly higher in MNP patients with obesity at presentation.

**Table 3.** Comparison of patients with hypertension at presentation.

	Hypertension				
Parameters	*n*	Yes	*n*	No	*p* Value
Age (years)	343	56 (18:87)	607	41 (18:82)	**<0.001** ^a^
Comorbidities					
Diabetes mellitus					
Yes	337	79 (23.4%)	619	27 (4.4%)	**<0.001^b^**
No		258 (76.6%)		592 (95.6%)	
Systolic blood pressure (mm/Hg)	300	140 (90:210)	560	120 (13:180)	**<0.001** ^a^
Diastolic blood pressure (mm/Hg)	300	80 (53:130)	560	80 (40:130)	**<0.001** ^a^
Biochemistry					
Glucose (mg/dL)	304	97.5 (61:485)	539	90 (44:991)	**<0.001** ^a^
Blood urea nitrogen (mg/dL)	320	18 (1:171)	588	13 (3:84)	**<0.001** ^a^
Creatinine (mg/dL)	325	0.9 (0.4:11.1)	589	0.8 (0.2:7.8)	**<0.001** ^a^
CKD-EPI eGFR (mL/min/1.73 m^2^)	325	83.70 (3.90:142.70)	582	109.50 (6.80:191.60)	**<0.001** ^a^
Urine protein(mg/24 h)	296	5744.5 (60:25,250)	522	6000 (3:31,185)	0.286^a^
Uric acid (mg/dL)	287	6.2 (2.7:11.8)	511	5.4 (2:11.9)	**<0.001** ^a^
Total cholesterol (mg/dL)	282	252 (88:694)	495	294 (100:721)	**<0.001** ^a^
Triglyceride (mg/dL)	284	192 (53:914)	501	202 (40:999)	0.793^a^
HDL-cholesterol (mg/dL)	269	48 (16:131)	456	52 (14:149)	**0.002** ^a^
LDL-cholesterol (mg/dL)	261	167 (35:457)	486	183.5 (14:799)	**<0.001** ^a^
Total protein (g/dL)	288	5.4 (3:10.2)	518	5.1 (2.8:9.6)	**0.007** ^a^
Albumin (g/dL)	307	2.8 (1:5.6)	569	2.5 (0.9:5)	**0.001** ^a^
Hemoglobin (g/dL)	309	12.6 ± 1.9	561	13.5 ± 1.8	**<0.001^c^**
Hematocrit (%)	300	38 (22:52)	548	40 (15:86)	**<0.001** ^a^
Histopathology					
Mesangial proliferation					
Yes	324	110 (34%)	578	186 (32.2%)	0.587^b^
No		214 (66%)		392 (67.8%)	
Interstitial inflammation					
Yes	320	209 (65.3%)	594	308 (51.9%)	**<0.001^b^**
No		111 (34.7%)		286 (48.1%)	
Interstitial fibrosis					
Absent	328	141 (43%)	593	355 (59.9%)	**<0.001^b^**
Grade 1 (<25%)		132 (40.2%)		187 (31.5%)	
Grade 2 (25–50%)		41 (12.5%)		40 (6.7%)	
Grade 3 (>50%)		14 (4.3%)		11 (1.9%)	
Vascular changes					
Yes	326	163 (50%)	592	153(25.8%)	**<0.001^b^**
No		163 (50%)		439(74.2%)	
Tubular atrophy					
*Absent*	328	127 (38.7%)	600	357 (59.5%)	**<0.001^b^**
Grade 1 (<25%)		151 (46%)		216 (36%)	
Grade 2 (25–50%)		39 (11.9%)		20 (3.3%)	
Grade 3 (>50%)		11 (3.4%)		7 (1.2%)	
Number of glomerular changes					
Yes	207	9 (4.3%)	348	16 (4.6%)	0.891^b^
No		198 (95.7%)		332 (95.4%)	
Total glomeruli in biopsy sample	336	15 (1:98)	608	15 (1:75)	0.345^a^
Global sclerotic	320	1 (0:24)	570	0 (0:18)	**<0.001** ^a^
Segmental sclerotic	279	0 (0:18)	529	0 (0:17)	0.381^a^
Crescentic glomeruli	278	0 (0:5)	520	0 (0:10)	0.998^a^

^a^Mann–Whitney U test; ^b^Chi-squared test; ^c^Independent samples t-test.

Bold values are statistically significant at *p* < 0.05.

### Comparison of patients according to eGFR values

The patients with eGFR >60 mL/min/1.73 m^2^ (we described as normal eGFR group) were significantly older and had higher diabetes and HT rates as comorbid conditions. The decreased eGFR group (≤60 mL/min/1.73 m^2^) had significantly higher systolic and diastolic blood pressure. The lipid profile abnormalities were more common in the decreased eGFR group compared with the normal eGFR group. Moreover, the mean serum albumin level was significantly lower in the normal eGFR group. On the other hand, the decreased eGFR group was significantly more anemic than the normal eGFR group.

Regarding pathological findings, the decreased eGFR group significantly had more global and segmental sclerotic glomeruli compared with the normal eGFR. Mesangial proliferation (39%), interstitial inflammation (75.2%), interstitial fibrosis (65.3%), and tubular atrophy (69.5%) were significantly more common in the decreased eGFR group compared the normal eGFR. Furthermore, there was no difference between the two groups in terms of immunofluorescence microscopy findings (Table 4).

**Table 4.** Comparison of patients with eGFR values below and over 60 mL/min.

Parameters	Patients with eGFR <60 (*n* = 155)	Patients with eGFR >60 (*n* = 774)	*p* Value
Age (years)	55.3 ± 13.2	44.7 ± 13.8	**<0.001** ^†^
Comorbidities			
Hypertension (*n* = 906)	99 (64.7%)	225 (29.9%)	<0.001*
Diabetes mellitus (*n* = 905)	25 (16.4%)	78 (10.4%)	0.036*
Systolic blood pressure (mm/Hg)	137.5 (122.5–148.6)	130 (120–140)	**<0.001** ^§^
Diastolic blood pressure (mm/Hg)	82.5 (73.5–91.5)	80 (70–83)	**0.001** ^§^
Biochemistry			
Glucose (mg/dL)	95.5 (86.3–107)	93 (85–104)	**0.024** ^§^
Blood urea nitrogen (BUN) (mg/dL)	31 (25–37.5)	15 (11–18)	**<0.001** ^§^
Urine Protein(mg/24 h)	6917 ± 441	7169 ± 259	0.651
Uric acid (mg/dL)	6.9 ± 1.7	5.7 ± 1.6	**<0.001** ^†^
Total cholesterol (mg/dL)	259 (186–322)	280 (228–335)	**<0.001** ^§^
Triglyceride (mg/dL)	182 (128–243)	209 (145–321)	**0.010** ^§^
HDL-cholesterol (mg/dL)	54 (39–72)	50 (41–59)	0.006^§^
LDL-cholesterol (mg/dL)	161 (131–210)	175 (137–237)	**<0.001** ^§^
Total protein (g/dL)	5.6 ± 1.0	5.3 ± 1.1	**0.002** ^†^
Albumin (g/dL)	3.0 ± 0.8	2.7 ± 0.9	**0.003** ^†^
Hemoglobin (g/dL)	11.8 ± 1.9	13.5 ± 1.7	**<0.001** ^†^
Hematocrit (%)	34 (31.3–37.5)	40 (37–44)	**<0.001** ^§^
Histopathology *n* (%)			
Mesangial proliferation (*n* = 878)	57 (39.0%)	222 (30.3%)	0.041*
Interstitial inflammation (*n* = 891)	112 (75.2%)	377 (50.8%)	<0.001*
Interstitial fibrosis (*n* = 892)			
Absent	52 (34.7%)	438 (59.0%)	<0.001*
Grade 1 (<25%)	55 (36.7%)	249 (33.6%)	
Grade 2 (25–50%)	32 (21.3%)	43 (5. 8%)	
Grade 3 (>50%)	11 (7.3%)	12 (1.6%)	
Vascular changes (*n* = 890)	85 (58.2%)	217 (29.2%)	<0.001**
Tubular atrophy (*n* = 897)			
Absent	46 (30.5%)	432 (57.9%)	<0.001*
Grade 1 (<25%)	66 (43.7%)	283 (37.9%)	
Grade 2 (25–50%)	32 (21.2%)	22 (2.9%)	
Grade 3 (>50%)	7 (4.6%)	9 (1.2%)	
Number of glomerular changes median (min–max)			
Total glomeruli in biopsy sample	13.5 (2–61)	17 (1–98)	**0.002** ^§^
Global sclerotic	2 (0–24)	0 (0–18)	**<0.001** ^§^
Segmental sclerotic	0 (0–8)	0 (0–18)	**<0.001** ^§^
Crescentic glomeruli	0 (0–2)	0 (0–5)	**0.036** ^§^

CKD-EPI: chronic kidney disease epidemiology study; GFR: glomerular filtration rate

*Chi-squared tests;

**Fisher’s exact test;

^§^Mann–Whitney U test;

^†^Independent samples t-test.

Post hoc analysis with Bonferroni correction showed the following: in interstitial fibrosis variable, significant difference was between grade 1 *vs.* grade 2, and grade 1 *vs.* grade 3, and absent *vs*. grade 1, in tubular atrophy variable: grade 1 *vs.* grade 2, and grade 1 *vs*. grade 3, and absent *vs.* grade 1. Bold values are statistically significant at *p* < 0.05.

For not to occupy too much space, we analyzed all variables in Tables 1 and 2 but presented only the variables that were significantly different between the groups.

The rate of patients with eGFR < 90 mL/min/1.73 m^2^ was 61.1%. Besides, at presentation, 5.5% of all study patients had a nephritic syndrome component.

### Comparison of patients according to age

The patients were grouped according to age as the older group (10.2%) who were older than 65 years and the younger group (89.8%) who were younger than 65 years. Diabetes and HT were more common in the older group as expected. Older patients had higher mean systolic blood pressure values and more frequently had pretibial edema at the time of the presentation. Older patients had significantly lower median eGFR (80.3 mL/min/1.73 m^2^, IQR, 58.4–90.5), lower serum hemoglobin, and a higher sedimentation rate compared with the younger group. On the other hand, serum albumin levels were comparable between the two groups.

The rate of global sclerotic glomeruli presence and the median number of global sclerotic glomeruli were significantly higher in the older group. Rates of interstitial inflammation, interstitial fibrosis, vascular changes, and tubular atrophy were significantly higher in the older group. Regarding the immunofluorescence findings, mild IgA deposition was significantly more common in the older group (Table 5).

**Table 5.** Comparison of disease characteristics between patients below and over 65 years.

Parameters	Patients <65 years (*n* = 880)	Patients ≥65 years (*n* = 100)	*p* Value
Comorbidities			
Hypertension (n = 950)	272 (31.9%)	71 (71%)	<0.001*
Diabetes mellitus (n = 949)	82 (9.6%)	25 (25%)	<0.001*
Pretibial edema (n = 896)	588 (72.9%)	76 (76%)	0.010*
Systolic blood pressure (mm/Hg)	130 (115–140)	137.5 (130–145)	**<0.001** ^§^
Biochemistry			
Glucose (mg/dL)	94 (86–104)	93 (83–112)	**0.001** ^§^
Blood urea nitrogen (BUN) (mg/dL)	15 (11–20)	21.5 (16–26)	**<0.001** ^§^
Creatinine (mg/dL)	0.8 (0.6–1.0)	0.9 (0.7–1.2)	**<0.001** ^§^
CKD-EPI estimated GFR (mL/min)	105.1 (83.1–119)	80.3 (58.4–90.5)	**<0.001** ^§^
Urine protein (mg/24 h)	7187 ± 241	6172 ± 446	0.168
Uric acid (mg/dL)	5.8 ± 1.7	6.6 ± 1.9	**<0.001** ^†^
Alanine aminotransferase (ALT) (U/L)	16 (13–21)	16.5 (12.3–19.8)	0.039^§^
Hemoglobin (g/dL)	13.3 ± 1.8	12.5 ± 2.2	**<0.001** ^†^
Hematocrit (%)	40 (36–44)	38.5 (32.3–42)	**0.003** ^§^
Sedimentation rate (mm/hour)	43.6 ± 27.5	51.1 ± 26.5	**0.034** ^†^
Histopathology *n* (%)			
Interstitial inflammation (*n* = 929)	459 (54.9%)	63 (67.7%)	0.020*
Interstitial fibrosis (*n* = 946)			
Absent	475 (56.7%)	34 (36.2%)	
Grade 1 (<25%)	274 (32.7%)	44 (46.8%)	0.003*
Grade 2 (25–50%)	68 (8.1%)	13(13.8%)	
Grade 3 (>50%)	21(2.5%)	3 (3.2%)	
Vascular changes (*n* = 932)	276 (32.9%)	44 (47.3%)	0.006*
Tubular atrophy (*n* = 938)			
Absent	458 (54.4%)	33 (34.4%)	0.002*
Grade 1 (<25%)	318 (37.8%)	53 (55.2%)	
Grade 2 (25–50%)	52 (6.2%)	6(6.3%)	
Grade 3 (>50%)	14(1.7%)	4 (4.2%)	
Number of glomerular changes median (min–max)			
Global sclerotic	0 (0–24)	2 (0–16)	**<0.001** ^§^
Immunofluorescence microscopy findings			
IgA (*n* = 890)			
Negative	670 (83.6%)	75 (84.3%)	
(+)	94 (11.7%)	5 (5.6%)	0.033**
(++)	25 (3.1%)	5 (5.6%)	
(+++)	12 (1.5%)	4 (4.5%)	

CKD-EPI: chronic kidney disease epidemiology study; GFR: glomerular filtration rate

*Chi-squared tests;

**Fisher’s exact test;

^§^Mann–Whitney U test;

^†^Independent samples t-test.

Post-hoc analysis with Bonferroni correction showed the following: in interstitial fibrosis variable, significant difference was between grade 1 *vs*. grade 2, and grade 1 *vs*. grade 3, and absent *vs*. grade 2, and absent *vs*. grade 3, in tubular atrophy variable: grade 1 *vs*. grade 2, and grade 1 *vs*. grade 3, and absent *vs.* grade 2, and absent *vs.* grade 3, in IgA staining: (+) *vs*. (+++), and (++) *vs*. (+++), and negative *vs*. (+++). Bold values are statistically significant at *p* < 0.05.

For not to occupy too much space, we analyzed all variables in Tables 1 and 2 but presented only the variables that were significantly different between the groups.

### Comparison of patients according to proteinuria

The patients were grouped according to proteinuria levels as nephrotic in whom daily proteinuria was above 3.5 g and as sub-nephrotic with proteinuria less than 3.5 g/d. There was no significant difference regarding age between the groups. Regarding gender, there was a significant predominance of the male gender in the nephrotic group. Sterile pyuria was significantly more frequent in the nephrotic group. In the nephrotic group, serum albumin level was significantly lower, and the rate of hyperlipidemia was markedly higher than in the sub nephrotic group, as expected (Table 6).

**Table 6.** The characteristics of patients according to presence of nephrotic range proteinuria.

Parameters	Patients with proteinuria <3500mg/day (*n* = 219)	Patients with proteinuria ≥3500 (*n* = 621)	*p* Value
Sex *n* (%)			
Female	106 (48.4%)	231 (37.2%)	0.004*
Male	113 (51.6%)	390 (62.8%)	
Pretibial edema (*n* = 810)	98 (47.8%)	503 (83.1%)	<0.001*
Pyuria (*n* = 796)	16 (8.1%)	85 (14.2%)	0.035*
Biochemistry			
Total cholesterol (mg/dL)	234 (105–271)	286 (234–351)	**<0.001** ^§^
Triglyceride (mg/dL)	152 (123–211)	216 (147–324)	**<0.001** ^§^
LDL-cholesterol (mg/dL)	143 (118–168)	189 (142–247)	**<0.001** ^§^
Total protein (g/dL)	6.1 ± 1.0	5.1 ± 1.0	**<0.001** ^†^
Albumin (g/dL)	3.4 ± 0.9	2.6 ± 0.7	**<0.001** ^†^
Alanine aminotransferase (ALT) (U/L)	16 (13.5–23)	16 (13–20.3)	**0.011** ^§^
Calcium (mg/dL)	9.0 ± 0.7	8.5 ± 0.8	**<0.001** ^†^
Hemoglobin (g/dL)	12.8 ± 1.9	13.3 ± 1.8	**0.002** ^†^
Hematocrit (%)	39 (35.5–43.5)	40 (36–44)	**0.003** ^§^
Duration of membranous nephropathy (months)	38 (9–80)	23 (7–54.3)	**0.010** ^§^
Histopathology *n* (%)			
Mesangial proliferation (*n* = 791)	74 (37%)	170 (28.8%)	0.033*
Interstitial inflammation (*n* = 803)	93 (45.6%)	342 (57.1%)	0.004*
Number of glomerular changes median (min–max)			
Crescentic glomeruli	0 (0–10)	0 (0–2)	**0.006** ^§^
Fibrocellular crescents	0 (0–3)	0 (0–2)	**0.034** ^§^
Immunofluorescence microscopy findings			
IgG (*n* = 793)			
Negative	12 (5.9%)	26 (4.4%)	0.002*
(+)	24 (11.7%)	36 (6.1%)	
(++)	58 (28.3%)	124 (21.1%)	
(+++)	111 (54.1%)	402 (68.4%)	
C1q (*n* = 714)			
Negative	153 (84.5%)	480 (90.1%)	0.004**
(+)	16 (8.8%)	39 (7.3%)	
(++)	10 (5.5%)	5 (0.9%)	
(+++)	2 (1.1%)	9 (1.7%)	
Kappa (*n* = 487)			
Negative	28 (29.8%)	120 (30.5%)	0.025*
(+)	27 (28.7%)	63 (16%)	
(++)	17 (18.1%)	106 (27%)	
(+++)	22 (23.4%)	104 (26.5%)	

*Chi-squared tests;

**Fisher’s exact test;

^§^Mann–Whitney U test;

^†^Independent samples t-test.

Post hoc analysis with Bonferroni correction showed the following: in IgG staining, significant difference was between (+) *vs*. (++), and (++) *vs.* (+++), in C1q staining: between (+) *vs.* (++), and negative *vs*. (++), in Kappa staining: (+) *vs*. (++). Bold values are statistically significant at *p* < 0.05.

For not to occupy too much space, we analyzed all variables in Tables 1 and 2 but presented only the variables that were significantly different between the groups.

Anti-PLA2R antibodies were studied only in 138 (13.9%) patients. Clinical and laboratory findings were similar between anti-PLA2R positive and anti-PLA2R negative patients. However, regarding biopsy findings, vascular changes were more prominent in anti-PLA2R positive patients compared with anti-PLA2R negative patients.

## Discussion

The main findings of this study can be summarized by Ronco and Debiec [1] Mean age of presentation was around 50 years, and males constituted the majority of the patients [2]. Most patients (81.7%) presented with nephrotic syndrome symptoms. On the other hand, a sizeable minority of patients had mixed nephritic-nephrotic syndrome and nephritic syndrome findings. Glomerular filtration rate was reduced at the time of the diagnosis in 15.6% of the patients [3]. One-third of all patients had microscopic hematuria, whereas more than 10% had sterile pyuria [4]. Serum C3 and C4 complement levels were decreased in a minority of the patients [5]. In addition, biopsy findings were significantly worse in patients with a history of HT compared to in patients with a history of obesity.

Pathologically, the most common finding was diffuse thickening of glomerular capillary walls. Moreover, endocapillary proliferation and interstitial inflammation, though atypical for primary MNP, were present in 5.6 and 55.9% of the patients. Occasionally crescentic glomeruli were observed. The most strong granular deposition was observed for IgG and C3 complement. In addition, glomerular IgM and IgA deposition were observed in kidney biopsy samples in 25 and 16% of the patients, respectively.

In most series, the share of the MNP among primary nephrotic syndrome causes ranges between 20 and 37% [2]. This is the largest study yet evaluating patients with primary MNP in Turkey. First, Ozturk et al. performed a multicenter epidemiologic study in which they evaluated the demographic and clinical characteristics of primary glomerular diseases in Turkey in 2014 using the same TSN-GOLD Primary Glomerulonephritis Registry that we have used in this study. Primary MNP was the most common glomerular disease (28.8%) in this study [9]. The repeated analysis was published in 2020 with updated data. This time the prevalence of primary MNP (25.6%) was second only to IgA nephropathy [8].

Our study’s mean age and male-predominant gender distribution were in line with known characteristics of primary MNP published before. Besides, the great majority of the patients presented with nephrotic syndrome, including generalized edema, hypoalbuminemia, nephrotic range proteinuria, and dyslipidemia. However, some of our findings are worth to be discussed further. In the literature, renal function during disease presentation has been reported as normal in more than 90% of patients with primary MNP [14,15]. However, our results revealed a much larger portion of patients (16.7% with GFR <60 mL/min/1.73 m^2^, 61.1% with eGFR <90 mL/min/1.73 m^2^) with compromised kidney function at the presentation. This observation might reflect the relatively late presentation of patients in our study. Besides, at presentation, 5.5% of all study patients had a nephritic syndrome component.

Significant histopathological changes in patients with HT deserve consideration. Troyanov et al. demonstrated that the severity of tubulointerstitial and vascular damage found on light microscopy does predict renal survival. In addition to proteinuria level and basal GFR, which are the most important prognostic indicators for renal survival in MNP patients, HT can also be an important prognostic indicator [16]. In the Japanese study with the highest number of cases in the literature, HT and possibly related tubulointerstitial changes were found to be associated with poor renal prognosis [17]. Kidney damage due to HT is characterized by renal fibrosis, tubular atrophy, and glomerular sclerosis changes. HT may contribute to tubulointerstitial changes, which are seen in the course of progressive MNP in our cohort. Significant tubulointerstitial changes in hypertensive patients in our study may be related to their older age. However, the immune system activated due to MNP may worsen HT and adversely affect renal survival. It has been recognized that immune cells contribute to blood pressure elevation in several experimental models [18]. Although obesity-related glomerulopathy is a well-known pathological diagnosis, its relationship with MNP has not been reported in the literature [19]. No significant effect of obesity on renal pathology was demonstrated in biopsy findings.

Microscopic hematuria is generally much more common in primary MNP than is generally thought and varies between 30 and 50% at presentation [5,20]. Among our patients, the frequency of microscopic hematuria was 33%. Moreover, 13% of the patients also had sterile pyuria. Interestingly, 3.7 and 1.7% of the patients had reduced serum levels of C3 and C4, respectively. Only six patients had reduced serum levels of both complement proteins simultaneously.

Our results were in agreement with the published literature with respect to the presence of common histopathologic findings, such as increased capillary basal membrane thickness. However, a significant proportion of our patients had mesangial proliferation (32.8%) and interstitial inflammation (45.4%), which are regarded as atypical findings in pure primary MNP. Furthermore, 5.6% of the biopsy samples, endocapillary proliferation was evident, which is highly unusual for primary MNP and should suggest alternative diagnoses. Since this was a retrospective and multicenter study, we did not have the opportunity to reevaluate the cases that had these unusual biopsy findings. Despite the efforts of individual centers, some secondary cases of MNP might have been missed out. Expectedly, interstitial fibrosis, tubular atrophy, and vascular changes were more prevalent in patients with reduced eGFR values. In a similar vein, these aforementioned changes were also significantly more common in patients older than 65 years.

Usually, the most remarkable staining in immunofluorescence microscopy in primary MNP is observed with IgG and C3 complement [21]. In contrast to primary MNP, secondary MNP exhibits deposition of C1q in glomerular capillary walls. In primary MNP, since IgG4 is incapable of binding C1q, an alternative complement cascade cannot be activated [21,22]. However, in small studies with sensitive assays of complement detection, a trace amounts of C1q deposition have been reported in patients with primary MNP [23,24]. Our results showed that the most prominent staining in immunofluorescence microscopy was with IgG and C3 complement. Nevertheless, in 11.6% of the biopsy samples C1q staining was positive. Of biopsy samples stained with C1q, only 13.8% showed a strong staining pattern. C1q staining frequency was not different in patients with reduced eGFR compared with patients who had eGFR > 60 mL/min/1.73 m^2^. Interestingly, patients with sub nephrotic proteinuria had significantly more common C1q staining compared to patients with nephrotic range proteinuria (15.5 *vs.* 9.9%).

Similarly, in primary MNP, staining with IgM and IgA is commonly seen but with low intensity. Dominant deposition of these immunoglobulins should suggest the presence of secondary MNP [25]. Our results demonstrated that in 25.4 and 16.3% of patients, glomeruli capillary walls were stained with IgM and IgA, respectively. However, of these patients, only 8.8 and 10.9% had strong staining with IgM and IgA, respectively. Although these patients are considered primary, they should be followed closely in terms of secondary diseases.

Some limitations of this study should be mentioned. First of all, the design of the study was retrospective. The multicenter design of the study might have resulted in some heterogeneity, particularly in histopathological findings. Second, only a minority of study patients had anti-PLA2R antibody test. This might have contributed to relatively atypical histopathological findings of our study as rendering distinguishing primary forms of MNP from the secondary causes more difficult. One of the most important shortcomings of our study is the lack of data on both conservative and immunosuppressive treatments applied to the patients, as well as long-term follow-up data. However, considering the possible effects of regional, national and racial differences, it is obvious that this study fills a gap in the literature.

## Conclusion

In conclusion, this is the most extensive MNP study conducted in Turkey. Our results revealed some unexpected findings, such as endocapillary proliferation, crescent formation, and low serum complement levels in some patients. On the other hand, major forms of disease presentation and classical light microscopy and immunofluorescence microscopy findings of kidney biopsy samples were largely in line with previously published research.

## Data Availability

The datasets used and/or analyzed during this study are available from the corresponding author on reasonable request. Trends in frequency of MNP in Turkey from 2009 to 2019.

## Abbreviations

TSN-GOLD: Glomerular Diseases Working Group of the Turkish Society of Nephrology
MNP: Membranous nephropathy
CKD-EPI: Chronic Kidney Disease Epidemiology Collaboration
HPF: High power field
IQR: Interquartile range
PLA2R: Anti-phospholipase A2 receptor
eGFR: estimated glomerular filtration ratio
